# Supplementary material for: Sex Differences in the Expression of Cardiac Remodeling and Inflammatory Cytokines in Patients with Obstructive Sleep Apnea and Atrial Fibrillation
Source: Biomedicines. 2024 May 23;12(6):1160. doi: 10.3390/biomedicines12061160 (PMC11200694; doi:10.3390/biomedicines12061160)
Supplement: Supplementary file 1 [file biomedicines-12-01160-s001.zip › biomedicines-3010020-supplementary.pdf]

Supplemental Table S1. The correlation between age and echocardiographic parameters and also gene expression of biomarkers by linear regression.

Variable	*B* (*SE*)	β	t	P value
LA (mm)	0.844 (0.623)	0.421	1.354	0.213
LVEF (%)	-0.024 (0.341)	-0.021	-0.069	0.947
E/e'	-1.163 (0.881)	-0.414	-1.320	0.223
IL-1β	-3.667 (6.356)	-0.182	-0.577	0.580
GJA1	-3.916 (5.747)	-0.200	-0.682	0.515

Adjust R^2: -0.016

Supplemental Table S2. The correlation between BMI and echocardiographic parameters and also gene expression of biomarkers by linear regression.

Variable	B (SE)	β	t	P value
LA (mm)	-0.136 (0.259)	-0.170	-0.525	0.614
LVEF (%)	-0.071 (0.142)	-0.161	-0.499	0.632
E/e'	0.588 (0.366)	0.524	1.609	0.146
IL-1β	1.425 (2.639)	0.177	0.540	0.604
GJA1	2.540 (2.386)	0.324	1.065	0.318

Adjust R^2: -0.096

Supplemental Table S3. The predictor of AF occurrence in OSA patients before and after age and BMI adjustment by logistic regression.

	Unadjusted			Adjusted age			Adjusted BMI			Adjusted age and BMI		
Variable	OR	95% CI	*P* value	OR	95% CI	*P* value	OR	95% CI	*P* value	OR	95% CI	*P* value
ECHO												
LA(mm)	1.298	1.058-1.591	0.012	1.246	1.002-1.549	0.048	1.305	1.055-1.613	0.014	1.259	1.003-1.581	0.047
LVEF(%)	0.900	0.796-1.018	0.093	0.899	0.793-1.018	0.094	0.894	0.784-1.020	0.096	0.895	0.784-1.021	0.099
E/e’	1.425	1.036-1.960	0.030	1.489	1.058-2.095	0.023	1.558	1.070-2.269	0.021	1.601	1.083-2.367	0.018
Gene												
IL-1β	0.617	0.147-2.590	0.510	0.706	0.157-3.169	0.650	0.703	0.172-2.876	0.624	0.776	0.178-3.386	0.735
GJA1	3.732	1.317-10.572	0.013	3.809	1.322-10.895	0.013	4.412	1.418-13.726	0.010	4.371	1.418-13.474	0.010
Age	-	-	-	1.049	0.948-1.160	0.359	-	-	-	1.040	0.938-1.153	0.453
BMI	-	-	-	-		-	0.861	0.651-1.140	0.296	0.877	0.661-1.162	0.359
